# Autoregulatory “Multitasking” at Endothelial Cell Junctions by Junction-Associated Intermittent Lamellipodia Controls Barrier Properties

**DOI:** 10.3389/fphys.2020.586921

**Published:** 2021-01-06

**Authors:** Jochen Seebach, Nadine Klusmeier, Hans Schnittler

**Affiliations:** Institute of Anatomy and Vascular Biology, Westfälische Wilhelms-Universität Münster, Münster, Germany

**Keywords:** VE-cadherin, JAIL, actin, permeability, junction dynamics, ARP2/3 complex

## Abstract

Vascular endothelial cell (EC) junctions are key structures controlling tissue homeostasis in physiology. In the last three decades, excellent studies have addressed many aspects of this complex and highly dynamic regulation, including cell signaling, remodeling processes of the proteins of tight junctions, adherens junctions, and gap junctions, the cytoskeleton, and post-transcriptional modifications, transcriptional activation, and gene silencing. In this dynamic process, vascular endothelial cadherin (VE-cadherin) provides the core structure of EC junctions mediating the physical adhesion of cells as well as the control of barrier function and monolayer integrity via remodeling processes, regulation of protein expression and post-translational modifications. In recent years, research teams have documented locally restricted dynamics of EC junctions in which actin-driven protrusions in plasma membranes play a central role. In this regard, our research group showed that the dynamics of VE-cadherin is driven by small (1–5 μm) actin-mediated protrusions in plasma membranes that, due to this specific function, were named “junction-associated intermittent lamellipodia” (JAIL). JAIL form at overlapping, adjacent cells, and exactly at this site new VE-cadherin interactions occur, leading to new VE-cadherin adhesion sites, a process that restores weak or lost VE-cadherin adhesion. Mechanistically, JAIL formation occurs locally restricted (1–5 μm) and underlies autoregulation in which the local VE-cadherin concentration is an important parameter. A decrease in the local concentration of VE-cadherin stimulates JAIL formation, whereas an increase in the concentration of VE-cadherin blocks it. JAIL mediated VE-cadherin remodeling at the subjunctional level have been shown to be of crucial importance in angiogenesis, wound healing, and changes in permeability during inflammation. The concept of subjunctional regulation of EC junctions is strongly supported by permeability assays, which can be employed to quantify actin-driven subjunctional changes. In this brief review, we summarize and discuss the current knowledge and concepts of subjunctional regulation in the endothelium.

## Introduction

The inner surface of blood vessels is covered by a thin monolayer of endothelial cells (ECs) whose total area in the human body is estimated to be 4000–7000 m^2^ (Aird, 2007). The vascular endothelium features organ- and vascular segment-specific phenotypes as verified in the pioneering work of Simionescu et al. (1975, 1976b). A central task of the endothelium is, apart from regulating blood pressure and organ perfusion, the control of exchange of water, gas, and solutes between the blood and the interstitium (regulated permeability). These functions are essential for the maintenance of tissue homeostasis and physiological adaptation processes. Control of permeability occurs by two mechanisms: a *transcellular pathway* controlled by transporters, transcytosis, or channels, and a *paracellular pathway* regulated by the dynamic opening and closing of cell junctions. Importantly, endothelial cell junctions have a key function during remodeling processes such as in the control of inflammatory responses, angiogenesis, wound healing, and tumor extravasation (Lampugnani et al., 2017; Duong and Vestweber, 2020). Tens of thousands of papers have been published on the vascular endothelium over the last few decades that have contributed to a fundamental understanding of the structure and regulation of endothelial cells and the endothelial cell junctions. However, most studies were performed on cell collectives that do not take into account locally restricted cell junction regulation and dynamics, both of which seems to be important for adaptational or remodeling processes of the junctions whose underlying mechanisms are not yet understood. The locally restricted transmigration of leukocytes and the relative movement of cells within a cell monolayer or in sheet migration are examples of the requirement for locally restricted dynamic junction regulation, as the overall monolayer integrity remains intact (see below). Those local phenomena cannot be adequately explained by general cell signaling mechanisms targeting the entire junctions. However, subcellular control of cell junctions and cell junction dynamics has remained an unsolved problem in cell biology for a long time, which was mostly due to inappropriate experimental and analytical techniques.

A significant improvement in live-cell microscopy techniques, the establishment of viral vectors for gene transduction in endothelial cells, and appropriate analytical software programs have contributed to a significant gain in knowledge with respect to cell junction dynamics, its local regulation, and the functional consequences for permeability. At this point the term *subjunctional* should be introduced: adjective refers to small, locally restricted areas of a few microns in length at the cell junction that can be locally opened or closed or undergo dynamic remodeling. It is therefore reasonable to assume that restricted local molecular interactions and signals at the cell contacts control this process. Indeed, work in recent years has revealed the first dynamic subjunctional structure, which were termed *junction-associated intermittent lamellipodia* (JAIL). JAIL are small, actin-driven plasma membrane protrusions of 1–5 μm in size that, in turn, directly drive the dynamics and remodeling of vascular endothelial cell adhesion molecules (VE-cadherin) via repeated formation of new VE-cadherin adhesions. A critical parameter controlling JAIL formation is the relative local VE-cadherin concentration (see below, under section “Subjunctional Regulation by JAIL Allows Multitasking Control of Endothelial Cell Junctions”). A local decrease in VE-cadherin facilitates JAIL formation while increasing amounts has inhibitory effects (Abu Taha et al., 2014; Cao et al., 2017). Since many different JAIL are constantly formed at the cell junctions, which also occur temporarily and at irregular intervals in time and space, we have postulated an autoregulatory mechanism for this phenomenon. Shortly, the discovery of this mechanism provides an extended concept of endothelial cell junction regulation that is able to explain subjunctional regulations required for inflammation, wound healing, angiogenesis, and shear stress adaptation. The functional impact of JAIL and the underlying mechanistic aspects are discussed in the following overview together with novel permeability assays that are able to detect local small differences in barrier function along endothelial cell junctions.

## Adherens Junctions in Vascular Endothelium

In contrast to the apicobasal order of tight, adherens, and gap junctions in the epithelium (Takeichi, 2014; Yonemura, 2017), the respective cell junctions in the endothelium are interwoven (Simionescu et al., 1976a). Regardless of the organ-specific diversity of endothelial cells and their cell contacts, adherens junctions are common to all endothelial phenotypes. They are characterized by the presence of endothelium-specific VE-cadherin (Suzuki et al., 1991; Lampugnani et al., 1995) and also by their close structural and functional association with the actin filament cytoskeleton via linker proteins such as catenins and others, as numerous studies on the physiology and pathophysiology of the endothelium have shown (Marcos-Ramiro et al., 2014; Schnittler et al., 2014; Taha and Schnittler, 2014; Garcia-Ponce et al., 2015; Schnoor, 2015; Zankov and Ogita, 2015; Alon and van Buul, 2017; Schnoor et al., 2017; Belvitch et al., 2018). VE-cadherin is a type-II calcium-dependent (Brasch et al., 2011) adhesion molecule that forms the backbone of adherens junctions and is thus expressed in all endothelial cells (Aird, 2006, 2007). The critical influence of VE-cadherin in endothelial cell biology has been demonstrated by studies in mice using blocking antibodies and genetic ablation of VE-cadherin that observed as a consequence an increase in vascular permeability and leukocyte transmigration but also an organ-specific heterogeneity (Corada et al., 2001; Frye et al., 2015). Furthermore, knockout of VE-cadherin expression in mice is lethal, which further underlines the important role of VE-cadherin in vascular development and homeostasis (Vittet et al., 1997; Carmeliet et al., 1999).

Vascular endothelial-cadherin is a single-spanning transmembrane protein with five extracellular repeats (EC1–EC5) at the amino terminus and a short cytosolic carboxy terminus that has binding sites for β-catenin associated with α-catenin. The extracellular domain of VE-cadherin connects adjacent cells by a homophilic, calcium ion-dependent interaction *via* the EC1/EC2 domains (Brasch et al., 2011), whereas the juxtamembrane region of the VE-cadherin-carboxy terminus binds p120 catenin, which is essential for the stabilization, internalization, and turnover of VE-cadherin (Reynolds, 2007; Vaughan et al., 2007; Komarova et al., 2017). The supramolecular organization of VE-cadherin at endothelial cell junctions has been sparsely studied. Investigations employing Stimulated Emission Depletion (STED) microscopy, however, showed clear evidence of a cluster-like structure. The VE-cadherin clusters were detected in several sizes and in two arrangements at the endothelial cell contacts: a linear arrangement of the clusters and a more planar arrangement, which was particularly visible at the overlapping cell junction areas. The size and number of clusters changed in response to external stimulation such as shear stress, which was accompanied by a functional modulation of the barrier function (Seebach et al., 2007).

There is a general consensus that the interdependent interaction between VE-cadherin and actin filaments is key in controlling endothelial permeability, barrier function, cell migration, and monolayer integrity, which are of critical importance in angiogenesis, inflammation, and wound healing. Either the β-catenin/α-catenin complex (Dejana and Vestweber, 2013) or, alternatively, the β-catenin/α-catenin/EPLIN complex are directly involved in connecting VE-cadherin to actin filaments. EPLIN (epithelial protein lost in neoplasm), which is an actin- and α-catenin-binding protein (Maul and Chang, 1999), has been shown to link actin filaments to adherens junctions in both epithelium and endothelium. In endothelium, EPLIN isoforms were shown to control actin dynamics in an isotype-specific manner, which directly impacts adherens junction dynamics and function (Abe and Takeichi, 2008; Chervin-Pétinot et al., 2012; Taha et al., 2019). It should also be mentioned that VE-cadherin is further associated with vimentin intermediary filaments via association with plakoglobin (γ-catenin); however, the functional role γ-catenin plays in this association is not completely understood. The few available studies on intermediate filaments and γ-catenin in endothelium indicate a role in junction stability, particularly during mechanical challenge by fluid shear stress of blood flow and leukocyte transmigration (Schnittler et al., 1997; Nottebaum et al., 2008; Muramatsu et al., 2017).

## Junction Associated Intermittent Lamellipodia (JAIL)

As outlined above, current issue in research into the regulation of EC junctions concerns the observation that the maintenance and restoration of monolayer integrity requires cellular junction dynamics that are limited to *subjunctional*, small cell contact areas of 1–5 μm rather than a central signal that triggers a uniform junction response (Cao and Schnittler, 2019). The currently best-understood subjunctional dynamics relate to actin-driven plasma membrane protrusions (comparable to classical lamellipodia) at EC junctions, which have been described independently by different authors (Doggett and Breslin, 2011; Hoelzle and Svitkina, 2012; Martinelli et al., 2013; Abu Taha et al., 2014; Adderley et al., 2015). The importance of these small (1–5 μm), actin-driven plasma membrane protrusions for the barrier function and monolayer integrity of ECs was elucidated by our research team. We showed that these transiently occurring membrane protrusions, which we named “junction-associated intermittent lamellipodia” (JAIL), led directly to new VE-cadherin adhesion sites (Abu Taha et al., 2014; Cao and Schnittler, 2019). There is evidence that JAIL-mediated VE-cadherin dynamics is autoregulated, which allows “multitasking activity” for recovery at different subjunctional sites at the same time.

Investigation of such a sophisticated dynamic mechanism requires advanced methodologies. In this case, we combined virus-mediated gene transfer of fluorescence-tagged proteins and fluorescence spinning-disk microscopy for time-lapse recordings and utilized divers software packages for the analyses. By expression of actin-binding and cell junction molecules such as VE-cadherin-mCherry or -EGFP, subunits of the ARP2/3 complex (EGFP-p20) or LifeAct-EGFP in ECs, our research team documented that the actin-driven protrusions lead directly to new VE-cadherin adhesions. This process occurs at a junction size between 1 and 5 μm in time frames of 5 min (Abu Taha et al., 2014; Seebach et al., 2015; Cao et al., 2017; Taha et al., 2019). Since JAIL formation is a continuous process that leads to new VE-cadherin adhesions, VE-cadherin dynamics is also subject to permanent remodeling. This process is the reason for the different VE-cadherin patterns observed along the endothelial cell junctions. Significant insight into this VE-cadherin remodeling by JAIL was also gained by studying and correlating cell density dependent cell motility/migration with JAIL-mediated VE-cadherin dynamics. Growing endothelial cultures show a very heterogeneous VE-cadherin pattern that is caused by the extended cell junction length. The different VE-cadherin patterns, which can be described as interrupted, linear or reticular, show different local concentrations of VE-cadherin. Accordingly, JAIL occur at sites where the Rel-VEcad-C is low, which is common in growing cultures with long cell contacts. Increasing cell density of up to 10^5^ cells/cm^2^ decreases the cell junction length and thus increases the number of areas with high Rel-VEcad-C. This leads to a decrease in JAIL frequency, JAIL size, and VE-cadherin dynamics occurs (Abu Taha et al., 2014). This shows that the JAIL activity depends on the local VE-cadherin pattern and the local VE-cadherin concentration, which is directly related to the length of the cell boundaries. The actin driven VE-cadherin dynamics showing this relationship is shown in the video annotated taken from Abu Taha et al. (2014) (herein Supplementary Movie 8). Documentation and interpretation of this mechanism is difficult using snapshots of fixed and immune-labeled cells, since the rapid spatiotemporal dynamics permanently changes the observed protein pattern. The best setup for investigating cellular dynamics is time-lapse recording setups using fluorescence and phase-contrast microscopy, since it allows visualization of rapidly occurring events. For time-lapse recordings the image acquisition frequency and the timeframe should be carefully selected. The reader is encouraged to view the accompanying video of JAIL-dependent VE-cadherin dynamics, taken from Cao et al. (2017) (herein Supplementary Movie 4).

## Subjunctional Regulation by JAIL Allows Multitasking Control of Endothelial Cell Junctions

**FIGURE 1:**
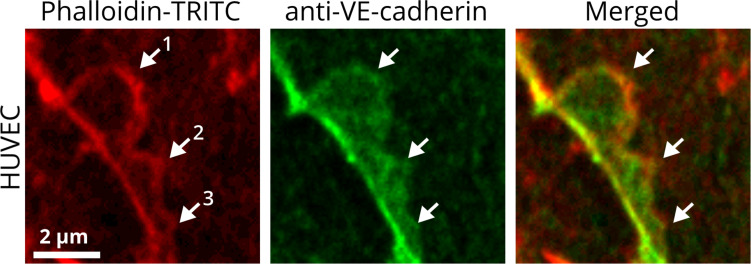
JAIL are subjunctional structures forming new VE-cadherin adhesion sites in the vascular endothelium. HUVEC cultures were treated with phalloidin-TRITC to label actin filaments and with anti-VE-cadherin to label cell junctions. JAIL of different sizes (1, 2, 3) are visible at EC junctions. Actin-driven JAIL induce VE-cadherin plaques directly, visible in the JAIL area that overlaps adjacent cells (merged). JAIL-mediated VE-cadherin dynamics occurs continuously and leads directly to new VE-cadherin adhesions. JAIL are formed at junctions where a local decrease in VE-cadherin-mediated adhesion appears, which may be spontaneous or caused by stimuli. The decrease in VE-cadherin is then a direct stimulus for JAIL formation to compensate for weak or absent VE-cadherin adhesion due to an increase in JAIL frequency and, thus, VE-cadherin-mediated cell adhesion. This interdependence is most likely of an autoregulatory nature. This mechanism also allows individual migration of cells within a cell monolayer while maintaining monolayer integrity. The dynamics of JAIL formation can be followed in the two movies.

One of the first examples of subjunctional regulation was described for leukocyte transmigration by actin-driven protrusions in plasma membranes, denoted “lateral lamellipodia.” Those lamellipodia have also been indicated to seal pores upon transcellular leukocytes transmigration. This occurs in a Rac- and actin-related protein (ARP)2/3 complex-dependent manner (Martinelli et al., 2013) and appears to be dependent on actin/myosin-mediated contractility (Heemskerk et al., 2016). While leukocyte transmigration is restricted to one subjunctional site, a more complex problem is related to subjunctional activity during cell growth and sheet migration. In particular, in a monolayer-forming cell sheet, each individual cell of the monolayer is surrounded by several adjacent cells and accordingly requires at least the same number of bilateral cell interconnections. In growing cell cultures, each of the individual cells within the monolayer displays an individual cell migration path, leading to displacement of cells relative to each other. Relative cell displacements and migration become more complex during endothelial sheet migration, as occurs in angiogenesis and wound healing. Under these conditions, some cells even migrate in opposite directions (Cao et al., 2017; Taha et al., 2019); however, the entire monolayer remains intact. In particular, the interaction of two adjacent cells in a monolayer, might appear to occur at a length of several dozen microns. Therefore, it becomes obvious that a further reduction of junction remodeling is required to allow a relative cell displacement. The high resolution of phase-contrast microscopy with time-lapse recording reveals that a cell monolayer remains almost intact irrespective of the dynamics, which is obviously due to high membrane activities at the cell borders. Protrusions corresponding to JAIL occur simultaneously or with different time delays at different cell junction locations of the same cell. The presence of JAIL at EC junctions was also confirmed by other authors in mammalian ECs (Neto et al., 2018; Chrifi et al., 2019; Gomez-Escudero et al., 2019). Structures with dynamics comparable to that of JAIL were demonstrated in the developing vasculature of fish and were described as “junction-based lamellipodia” (Paatero et al., 2018). Mechanistically, the subjunctional decrease in the amount of VE-cadherin at distinct junction sites (e.g., by gap formation or change in cell shape) leads to activation of the Rac/Wave/WASP/ARP2/3 complex. This activation causes the formation of branched actin filaments that promote locally restricted protrusions of the plasma membrane (Abu Taha et al., 2014). These JAIL overlap adjacent cells and it is only in this area that interactions of VE-cadherin molecules can occur, which diffuse freely in the plasma membrane and form a structure known as a “VE-cadherin plaque (Figure 1).” Subsequently, after actin filaments have been depolymerized at the protrusion front [e.g., by EPLIN-a-mediated blockade of the ARP2/3 complex (Taha et al., 2019)], JAIL retract and VE-cadherin molecules of the plaques cluster together and become incorporated into junctions, thereby closing gaps or restoring insufficient/weak areas of cell junctions (Abu Taha et al., 2014).

Numerous JAIL arise simultaneously or form with overlapping timeframes, so knowing which regulatory principles underlie the formation of an individual JAIL is important. There is strong evidence that subjunctional JAIL-mediated VE-cadherin dynamics follow an autoregulatory mechanism, which, for the first time, demonstrates the possibility that endothelial contacts are controlled at different sites independently. Local formation of JAIL is dependent upon the subjunctional concentration of VE-cadherin, whereby a low level increases and a high level blocks JAIL formation, as demonstrated by overexpression of VE-cadherin in EC cultures (Abu Taha et al., 2014; Cao et al., 2017). For quantitative analyses of local JAIL formation, it is helpful to calculate the relative VE-cadherin concentration (Rel-VEcad-C), which is defined as the ratio between the local concentration of VE-cadherin (e.g., measured fluorescence intensity) along a given cell border and the corresponding length of the cell junction (Cao and Schnittler, 2019). This is where the cell shape comes into play. After application of vascular endothelial growth factor (VEGF) to confluent cell cultures, or under wound-healing conditions, or during angiogenesis in the developing retina, ECs change shape, which leads to an increase in cell-cell contact length within sheets of ECs. VE-cadherin expression in these cells remains unchanged, so cell elongation dilutes the given amount of VE-cadherin and leads to a decrease in Rel-VEcad-C at cell contacts. A decrease in the VE-cadherin concentration stimulates JAIL formation, which increases both cell junction and overall dynamics of the cells.

The mechanism of JAIL-induced VE-cadherin dynamics has been shown to be essential in randomized and polarized cell migration and to be observed in growing cell cultures, angiogenesis, wound healing, and during shear stress-induced morphologic adaptation of ECs (Cao et al., 2017; Taha et al., 2019). These studies showed that, during directed polarized cell migration, large JAIL occur at the migration front, whereas only small JAIL develop at the lateral borders to neighboring cells. Analyses of VE-cadherin dynamics by spinning-disk live-cell imaging in cultured ECs revealed the intermittent appearance of an interrupted VE-cadherin alternating with large JAIL-mediated VE-cadherin plaques, whereas the overall appearance of VE-cadherin at lateral junctions was faint but linear, which explained the formation of small JAIL at this site. Functionally, large JAIL developing at the leading cell pole direct and drive polarized cell migration whereas JAIL at the lateral junctions allow relative displacement of adjacent cells. This observation suggests that asymmetric JAIL dynamics are also involved in establishing cell polarity, although the underlying mechanisms of JAIL polarization are incompletely understood.

As shown for actin-mediated closure of micro-wounds (Martinelli et al., 2013), JAIL-mediated VE-cadherin dynamics close mediator-induced or spontaneously appearing intercellular gaps (Abu Taha et al., 2014; Seebach et al., 2015; Neto et al., 2018; Gomez-Escudero et al., 2019). This feature is relevant to maintaining overall endothelial integrity under resting conditions as well as during inflammation with increased permeability and leukocyte transmigration (Martinelli et al., 2013; Breslin et al., 2015; Seebach et al., 2015; Heemskerk et al., 2016). Junction remodeling under physiologic and pathologic conditions has been shown to involve several mechanisms, including activation of Rho-GTPases, PI 3-kinase, Src, PKC, actin/myosin contractility, myosin light-chain kinases and phosphatases, and the vascular endothelial phosphotyrosine phosphatase (VE-PTP) (Kuppers et al., 2014; Komarova et al., 2017; Duong and Vestweber, 2020).

The concept of junction control by subjunctional dynamics is in accordance with work that revealed N-WASP as an important regulator in the recovery of barrier function after thrombin stimulation (Rajput et al., 2013; Abu Taha et al., 2014; Seebach et al., 2015; Belvitch et al., 2017; Cao et al., 2017). N-WASP is a nucleation-promoting factor that activates ARP2/3 and controls actin dynamics (Schnoor, 2015; Pollard, 2016; Steffen et al., 2017; Svitkina, 2018). Dynamic structures comparable to JAIL were later demonstrated in developing vessels of the zebrafish (Paatero et al., 2018), and as local lamellipodia in cell culture (Breslin et al., 2015). These structures, together with the immunofluorescence microscopic identification of JAIL in the developing retina and in the yolk sack of the mouse (Cao et al., 2017), strongly suggest a general subjunctional regulation of endothelial cell contacts in cultured endothelial cells and *in vivo*. Since the paracellular transendothelial migration (TEM) of leukocytes is accompanied by a local opening of cell junctions and most likely a restricted loss of VE-cadherin-mediated adhesion, it would be challenging to find out whether local inhibition of JAIL might play a role in TEM, as was demonstrated for the increase in permeability induced by thrombin (Breslin et al., 2015; Seebach et al., 2015). The dissociation of VE-cadherin during TEM, however, has been shown to include targeted trafficking of the so-called lateral border recycling compartment (LBRC), a reticulum of interconnecting vesicle-like structures along the endothelial cell border that contains a pool of PECAM-1 necessary for effective TEM (Mamdouh et al., 2003; Gonzalez et al., 2016). Intriguingly, the recruitment of the LBRC depends on kinesin and the microtubule cytoskeleton for independent regulation of actin-dependent VE-cadherin adhesion between neighboring endothelial cells on the one hand and PECAM-1-mediated adhesion between endothelial cells and the transmigrating leukocyte on the other hand. Since the dissociation of VE-cadherin occurs downstream of the trafficking event, it is intriguing to speculate that the LBRC might be associated with local JAIL activity. A link between YAP/TAZ signaling and JAIL has been reported, further indicating the critical impact of this mechanism in angiogenesis (Neto et al., 2018). In summary, these data strongly support our hypothesis on subjunctional cell-contact dynamics and further underline the functional importance of JAIL. However, there are still many questions to be answered regarding the coordination between actin-driven protrusions such as JAIL, dynamics of actin filaments, VE-cadherin remodeling, and cell adhesion.

## Subjunctional Control of Endothelial Barrier Functions

Quantifying the organ- and vascular bed-specific characteristics of the endothelial barrier allows mechanistic insights into physiologic and pathologic regulation. In recent decades, several *in vitro* and *in vivo* methods have been developed. The most frequently used and common parameters to describe the integrity of the endothelial barrier *in vitro* are the permeability to: (i) tracer molecules described by the permeability coefficient (PE); (ii) ions expressed as the transendothelial electrical resistance (TEER or TER); (iii) water quantified by the hydraulic conductivity (LP) (Wegener and Seebach, 2014). These assays have contributed significantly to the understanding of the mechanisms governing the function of the endothelial barrier. However, immunofluorescence microscopy using VE-cadherin antibodies of ECs treated with permeability-enhancing agents such as histamine, tumor necrosis factor-α, or thrombin show a heterogeneous distribution of VE-cadherin clusters at cell junctions described variously as “interrupted,” “linear,” “reticular,” and “invaginated” [(Cao and Schnittler, 2019), and references therein]. These data, together with results of fluorescence-based live-cell microscopic studies, led to the hypothesis that endothelial barrier function is related to dynamic changes at a subjunctional level (Cao and Schnittler, 2019). Knowledge about the dynamic reorganization of junctional proteins has benefited largely from the rapid development of innovative live-cell imaging methods in recent decades. However, knowledge about the resulting effects on local barrier function at cellular and subjunctional levels is still limited. The ability to determine local permeability, particularly in sheet-forming cell layers, would further enlighten many phenomena accompanying the transient loss of intercellular junctions as observed in cell division, apoptosis, or regenerative and repair processes. Indeed, permeability assays have been developed that can be used to obtain spatial resolution at the cellular (and even subjunctional) level, as smmarized in Table 1 and describd in more detail below.

**TABLE 1:** Assays for the detection of local permeability in sheet-forming cell layers.

Assay	Method	Applied to	Spatial Resolution	Living Cells	Time Resolution	References
Ion Conductivity	Microelectrodes	Epithelium	μm	Yes	ms to sec	Frömter and Diamond, 1972; Gitter et al., 2000; Florian et al., 2002
Transport Assay	Immobilized Fluorescence Tracer	Endothelium	mm	No	n.a.	Phelps and DePaola, 2000
Macroporous Assay	Collection of Tracer in Pores	Epithelium	μm	Yes	n.d.	Michaelis et al., 2012
XPerT	Fluorescent Avidin/Biotin	Endothelium, Epithelium	μm	No	n.a.	Dubrovskyi et al., 2013; Song et al., 2016
DyMEB	TIRF Microscopy	Endothelium	μm	Yes	1 min	Klusmeier et al., 2019

*n.a., not available; n.d., not determined.*

## Assays Used to Detect Local Permeability Along Cell Junctions

For investigation of local permeability in cell layers, Frömter and Diamond established in the early 1970s a test of ionic conductivity based on microelectrodes that were used to scan the cell layer with a resolution of ∼1.5 μm. Improvements of these methods then provided the possibility to determine permeability kinetics after single-cell defects caused by, for example, apoptosis or mechanical manipulation (Gitter et al., 2000; Florian et al., 2002). These studies were the first to describe local changes in permeability in EC layers. In recent decades, fluorescence-labeled macromolecules of different sizes have been used as tracers to determine quantitatively local permeability in epithelial and EC layers by various methods. In particular, Phelps and DePaola (2000) established an assay for the quantification of local flow-induced permeability at EC monolayers. For this assay, cells are cultured on a filter membrane and transferred to an agarose layer. Fluorescein isothiocyanate (FITC)-dextran molecules passing through the cell layer accumulate topically in the agarose layer and can be measured quantitatively. Even though this assay recognizes permeability of cell collectives in a lateral resolution of 1 mm, it is able to detect local differences in endothelial barrier function in response to disturbed flow profiles.

Development of the XPerT assay offered a significant increase in spatial resolution (Dubrovskyi et al., 2013). In this assay, ECs are grown on biotin-labeled solid substrates. FITC-labeled avidin is used as a tracer, which binds with high affinity to the immobilized biotin. After washing and fixing the samples, local differences in permeability are detected by fluorescence microscopy with a resolution of a few micrometers. Application of this assay revealed subjunctional changes in permeability triggered by mechanical stimulation or pro-inflammatory agonists (Dubrovskyi et al., 2013). This assay is based on endpoint measurement, so analyses of dynamics are not possible. Other assays also use fluorescence-coupled molecules as tracers which, after passing through a cell layer, accumulate in a macroporous silicon chip composed of circular pores of diameter 1.3 μm (Michaelis et al., 2012). Pores are arranged so close together that one cell covers many pores, which determines the local resolution. Thus, paracellular permeability and transcellular transport through the cell body can be detected. In addition to determination of local paracellular permeability, the method is very well suited for characterization of the basic transport routes of substances through a cell monolayer provided that the transport substance is fluorescence labeled.

**FIGURE 2:**
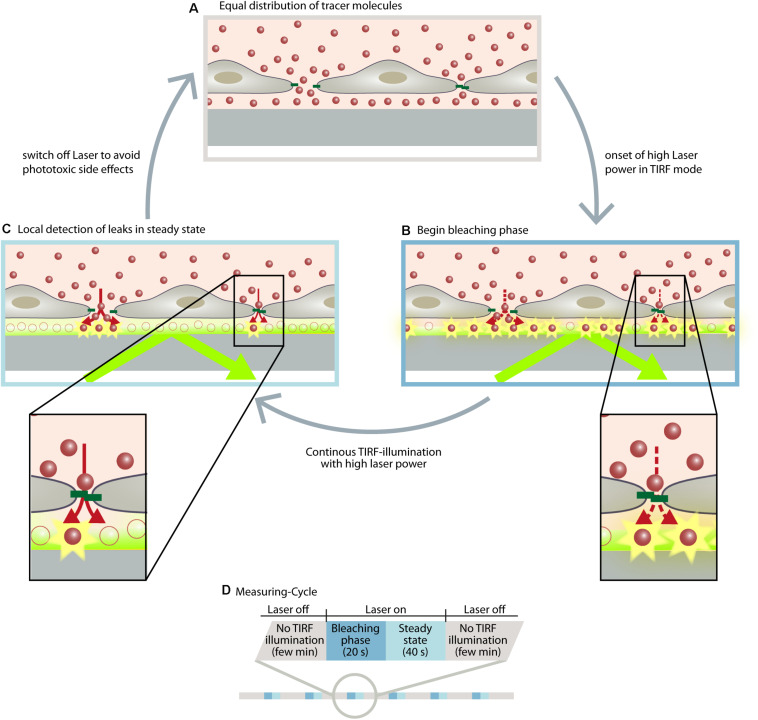
The DyMEB assay was designed to analyze the dynamics of barrier function at the subjunctional level in cultures of confluent endothelial cells. **(A)** Endothelial cells were cultured on glass-bottomed dishes suitable for TIRF microscopy, and a fluorescence-labeled marker molecule (e.g., Atto565-Dextran) was added to the culture medium. Due to the small volume of the basal compartment, an equal distribution between apical and basal sides of the cell is reached within a few minutes, even in confluent cell layers. **(B)** Due to TIRF illumination, fluorescence-labeled molecules are bleached at the glass/medium interface due to the high power of the laser. This generates a concentration gradient of fluorescent-labeled molecules between the apical and basal compartment, which continues the diffusion (dotted arrows). **(C)** The diffusion rate and the bleaching rate reach a steady state in which regions with higher permeability show a greater fluorescence intensity than regions with lesser permeability. **(D)** Illustration of the measuring cycles to avoid phototoxic effects. Furthermore antioxidants are added to the medium and the measurements are carried out at intervals of ∼2 min so that the reactive oxygen species formed by the TIRF illumination can be inactivated or degraded. During this interval other microscopic techniques (DIC, phase contrast, LSM) can be applied to analyze structural parameters of the cells. For further detailed information, see Klusmeier et al. (2019).

With the discovery of subjunctional dynamics, we aimed to establish a permeability assay that could detect permeability dynamics at the subjunctional level in the vascular endothelium. Therefore, the Seebach research team at the Institute of Anatomy and Vascular Biology (Münster, Germany) developed the “dynamic measurement of local endothelial barrier function in living endothelial cells” (DyMEB) assay. Fluorescence-tagged tracers of different molecular weight pass an EC layer at particular subjunctional sites and are detected by total internal reflection fluorescence (TIRF) microscopy. In TIRF mode, an evanescent field of ≤100 nm in the z-plane is established and fluorescence can be detected in this field only. The assay achieves a subjunctional lateral resolution of ∼15 μm with a high temporal resolution of 1 min (Klusmeier et al., 2019). More details of the DyMEB assay are illustrated in Figure 2.

**FIGURE 3:**
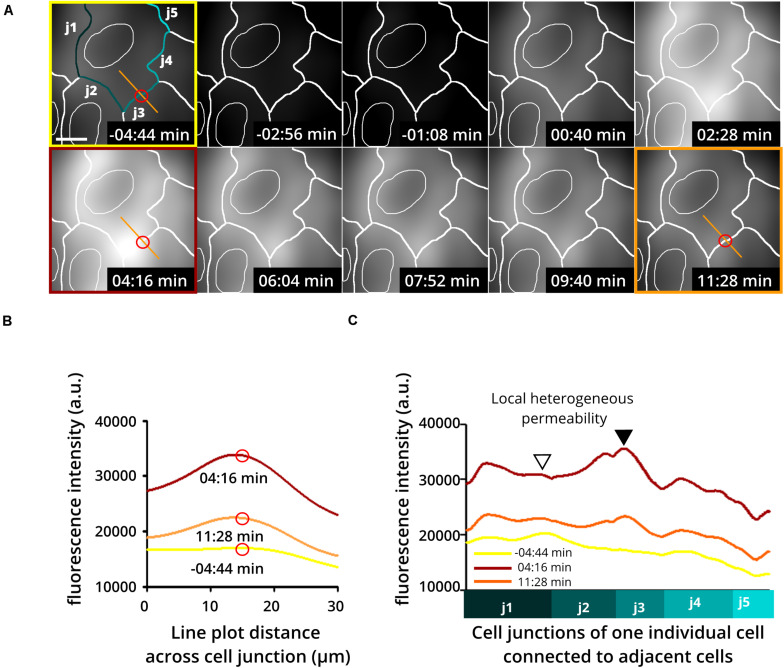
Heterogeneous and dynamic change in barrier function at the subjunctional level as shown upon histamine application to confluent endothelial cell cultures. Histamine is a proinflammatory product of mast cells and basophils known to increase endothelial permeability. Here, human umbilical vein endothelial cells (HUVEC) were treated with histamine to determine changes in endothelial permeability. **(A)** Time-lapse recording using the DyMEB assay combined with DIC images (not shown) taken alternately with TIRF microscopy allow an estimation of cell junctions (white lines). (Upper 3 left-hand images) Prior to histamine application, background fluorescence was low. Application of histamine (100 μM) increased the detectable fluorescence in a transient and heterogeneous pattern corresponding to the indicated cell junctions as a result of a heterogeneous increase in permeability. Scale bar, 10 μm. **(B)** Line plots taken from images shown in **(A)** at different time points as indicated by the red circle and orange line. **(C)** Line plots of junction-related fluorescence intensity at different time points for locations j1 to j5 in **(A)**. The different levels of brightness document the heterogeneous local permeability.

Application of this assay has already shown the extraordinary heterogeneity and dynamics of subjunctional permeability in cultures of resting confluent ECs. Furthermore, evaluation of subjunctional changes in permeability due to the proinflammatory agonist histamine (Moy et al., 1993; Di Lorenzo et al., 2009; Wessel et al., 2014) indeed displayed heterogeneous subjunctional permeability between different cells and, remarkably, even along the junctions of one EC (Figure 3). Close evaluation revealed that some subjunctional sections remained unaffected, whereas other areas displayed rapidly increased fluorescence in the TIRF mode (Figure 3). This subjunctional activity was consistent with studies showing subjunctional RhoA activity (Szulcek et al., 2013), and regulation of gap closure has also been proposed for Rac1 (Martinelli et al., 2013; Timmerman et al., 2015; Cao et al., 2017). Furthermore, histamine induced a change in barrier function that was suggested to be dependent upon activation of Rac1 and RhoA (Wojciak-Stothard et al., 2001).

In summary, the spatiotemporal heterogeneity of endothelial permeability at rest and under stimulation strongly support the concept of subjunctional dynamics of cell junction proteins, as demonstrated for JAIL-mediated VE-cadherin. In addition, dynamic measurements of subjunctional permeability combined with differential interference contrast microscopy time-lapse imaging can be used to analyze cellular morphodynamics. Laser scanning live-cell microscopy of fluorescence-labeled molecules can be used to visualize protein dynamics. Application of both of these methods allows analysis and correlation of structural and functional changes at cell contacts. The mechanistic concept of subjunctional regulation of cell junctions opens up the possibility of “fine tuning” and autoregulating permeability during dynamic remodeling of a cell layer.

## Conclusion and Future Perspectives

The discovery of the subjunctional control of EC contacts explains several phenomena that were difficult to understand previously. One of the challenging questions relates to migration of individual cells within a confluent cell layer, such as that occurring in angiogenesis and in wound healing where, surprisingly, monolayer integrity remains largely intact. This phenomenon cannot be explained exclusively by the classical models of signaling mechanisms and pathways. The extended model of subjunctional autoregulatory control of cell contacts within the range of 1–5 μm has been demonstrated convincingly in recent years, at least for ECs. This model meets all requirements to explain migration of individual cells within a cell layer while maintaining monolayer integrity, and it also provides a concept for the mechanism of opening and closing of cell junctions due to transmigration of leukocytes or tumor cells. For dynamic analyses, combined application of morphologic methods involving optogenetic tools (Gautier et al., 2014; Karunarathne et al., 2015), such as light-sensitive kinases or GTPases (Guglielmi et al., 2016; Leopold et al., 2018), together with a permeability assay to analyze subjunctional dynamics opens further possibilities to investigate mechanisms of local regulation of the function of endothelial barriers. Challenging work in the future will be to determine how classical signaling mechanisms can interfere and target particular cell-junction sites to control the permeability of cell monolayers.

## Author Contributions

All authors listed have made a substantial, direct and intellectual contribution to the work, and approved it for publication.

## Conflict of Interest

The authors declare that the research was conducted in the absence of any commercial or financial relationships that could be construed as a potential conflict of interest.
